# Neutralization of Lipocalin-2 Diminishes Stroke-Reperfusion Injury

**DOI:** 10.3390/ijms21176253

**Published:** 2020-08-29

**Authors:** Guona Wang, Yi-Chinn Weng, I-Chen Chiang, Yu-Ting Huang, Yi-Chu Liao, Yi-Chun Chen, Cheng-Yuan Kao, Yu-Li Liu, Tsong-Hai Lee, Wen-Hai Chou

**Affiliations:** 1 Department of Biological Sciences, School of Biomedical Sciences, Kent State University, Kent, OH 44242, USA; wgngm001@gmail.com; 2 Center for Neuropsychiatric Research, National Health Research Institutes, Miaoli 35041, Taiwan; wengyc050706@nhri.edu.tw (Y.-C.W.); c061010@nhri.edu.tw (I.-C.C.); 130613@nhri.edu.tw (Y.-T.H.); ylliou@nhri.edu.tw (Y.-L.L.); 3 Institute of Population Health Sciences, National Health Research Institutes, Miaoli 35041, Taiwan; ycliao@nhri.edu.tw; 4 Department of Neurology, Chang Gung Memorial Hospital Linkou Medical Center and College of Medicine, Chang Gung University, Taoyuan 33305, Taiwan; asd108@cgmh.org.tw; 5 Immunology Research Center, National Health Research Institutes, Zhunan, Miaoli 35041, Taiwan; chengyuankao@nhri.edu.tw; 6 Stroke Center and Department of Neurology, Linkou Chang Gung Memorial Hospital and College of Medicine, Chang Gung University, Taoyuan 33305, Taiwan; thlee@adm.cgmh.org.tw

**Keywords:** Lipocalin-2, stroke, reperfusion injury, oxidative stress, immunotherapy, neutrophils

## Abstract

Oxidative stress is a key contributor to the pathogenesis of stroke-reperfusion injury. Neuroinflammatory peptides released after ischemic stroke mediate reperfusion injury. Previous studies, including ours, have shown that lipocalin-2 (LCN2) is secreted in response to cerebral ischemia to promote reperfusion injury. Genetic deletion of LCN2 significantly reduces brain injury after stroke, suggesting that LCN2 is a mediator of reperfusion injury and a potential therapeutic target. Immunotherapy has the potential to harness neuroinflammatory responses and provides neuroprotection against stroke. Here we report that LCN2 was induced on the inner surface of cerebral endothelial cells, neutrophils, and astrocytes that gatekeep the blood–brain barrier (BBB) after stroke. LCN2 monoclonal antibody (mAb) specifically targeted LCN2 in vitro and in vivo, attenuating the induction of LCN2 and pro-inflammatory mediators (iNOS, IL-6, CCL2, and CCL9) after stroke. Administration of LCN2 mAb at 4 h after stroke significantly reduced neurological deficits, cerebral infarction, edema, BBB leakage, and infiltration of neutrophils. The binding epitope of LCN2 mAb was mapped to the β3 and β4 strands, which are responsible for maintaining the integrity of LCN2 cup-shaped structure. These data indicate that LCN2 can be pharmacologically targeted using a specific mAb to reduce reperfusion injury after stroke.

## 1. Introduction

Ischemic stroke is a leading cause of mortality and adult disability worldwide [1]. Thrombolysis with intravenous tissue plasminogen activator (tPA) and mechanical thrombectomy are the only procedures approved by the FDA to treat acute ischemic stroke [2]. However, the therapeutic benefits of tPA 4.5 h after stroke onset are outweighed by the risks of hemorrhagic transformation. Thrombectomy can be performed up to 24 h after symptom onset, but is intended for ischemic strokes caused by a large-vessel occlusion and can only be operated in stroke centers with sufficient resources and expertise [3]. Due to these limitations, it is estimated that less than 10% of stroke patients benefit from thrombolytic therapy [4].

Although restoring cerebral blood flow is the goal of thrombolytic therapy, reperfusion itself may lead to the formation of cytotoxic reactive oxygen species (ROS), infiltration of peripheral immune cells, intense inflammatory responses, hemorrhagic transformation, and exacerbated cerebral infarction [5,6,7]. Neutrophils are the first surge of blood cells that arrive at ischemic brain tissues, and ultimately contribute to oxidative stress, breakdown of the blood–brain barrier (BBB), cerebral edema, and brain injury [8,9]. Migration of neutrophils from the bloodstream across the vascular endothelium to ischemic brain tissues is mediated by the local release of ROS, chemokines, and cytokines from the BBB.

Lipocalin-2 (LCN2), also known as siderocalin, 24p3, or neutrophil gelatinase-associated lipocalin (NGAL), is a 25 kDa protein that is stored in specific granules of neutrophils and secreted in response to oxidative stress and a variety of CNS injuries [10,11,12]. Plasma levels of LCN2 are significantly elevated in ischemic stroke patients with unfavorable modified Rankin scale scores [13,14], post-stroke infections [14], hemorrhagic transformations after tPA treatments [15], and cardiovascular mortality [16]. These findings suggest that LCN2 may be used as a biomarker to identify oxidative stress and predict the clinical outcomes of stroke patients [12,17]. Previously, we and others found that LCN2 was acutely induced after transient middle cerebral artery occlusion (tMCAo) [18,19]. Elevated levels of LCN2 protein were observed in mouse serum as early as one hour after tMCAo, peaked at 23 h post-tMCAo, and were reduced at 48 to 72 h post-tMCAo [19]. Although LCN2 was undetectable in uninjured mouse brains, it was induced in the cerebral endothelial cells, infiltrated neutrophils, and astrocytes in the ipsilateral hemispheres at 24 h after tMCAo. Cerebral infarction, neurological deficits, infiltration of neutrophils, and BBB permeability were diminished in LCN2 deficient mice after tMCAo [18,19]. Based on these results, we hypothesized that therapeutically targeting LCN2 would reduce stroke-reperfusion injury. Monoclonal antibodies are a fast-growing class of therapeutics because of their high specificity, minimal toxicity, and engineerable manufacturing processes [20,21]. LCN2-targeted immunotherapy may be an effective treatment because LCN2 is released in the extracellular space, where it is accessible to antibodies. Therefore, the current study investigated if LCN2 specific antibodies neutralized LCN2 and diminished neuroinflammation and neurodegeneration after ischemic stroke.

## 2. Results

### 2.1. LCN2 was Detected in Cerebral Endothelial Cells, Astrocytes, and Infiltrating Neutrophils in the Ipsilateral Hemisphere after tMCAo

**Figure 1:**
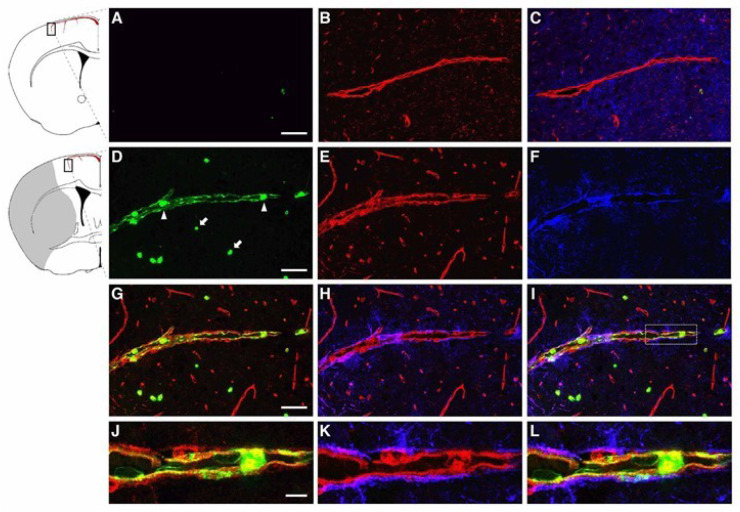
Immunolocalization of LCN2 in vascular endothelial cells in the ipsilateral cortex after transient middle cerebral artery occlusion (tMCAo). Brain slices isolated from naive mice (**A**–**C**) and at 23 h after tMCAo (**D**–**L**) were labeled with LCN2 antibody (green), tomato lectin (red, blood vessel), and GFAP antibody (blue, astrocyte). (**D**) Neutrophils detected within the blood vessel (arrowheads) and in ischemic brain parenchyma (arrows) labeled with LCN2 antibody (green). (**G**–**L**) Merged and amplified images showing the induction of LCN2 (green) on the inner surface of vascular endothelial cells (red) surrounded by astrocytic end-feet (blue). The shaded area in the inset indicates the infarcted region. Scale bars, 50 μm for the main images (**A**–**I**), and 10 μm for the amplified images (**J**–**L**).

**Figure 2:**
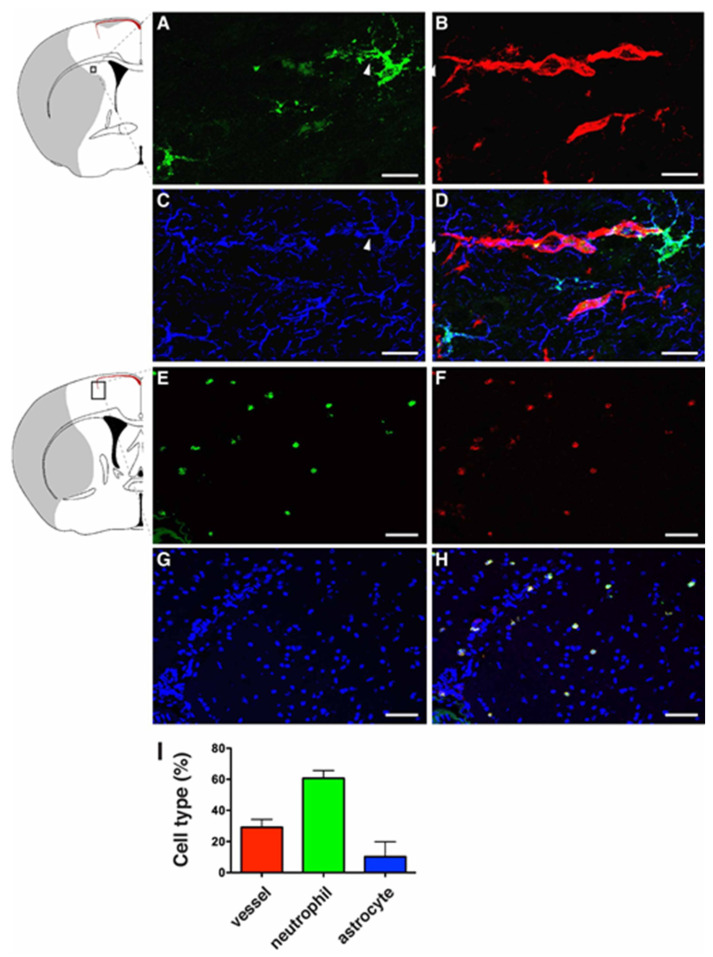
Immunolocalization of LCN2 in astrocytes and neutrophils in the ipsilateral cortex after tMCAo. Mouse brain slices isolated at 23 h after tMCAo were labeled with LCN2 antibody (green, **A**), Tomato Lectin (red, blood vessel, **B**), and GFAP antibody (blue, astrocyte, **C**). (**D**) Merged image showing the expression of LCN2 in an astrocyte whose end-feet encircle blood vessels (arrowheads). Brain slices isolated at 23 h after tMCAo were stained with antibodies recognizing LCN2 (green, **E**) and a specific marker for neutrophils (anti-Ly-6B.2 clone 7/4) (red, **F**). Nuclei were labeled with DAPI (blue, **G**). (**H**) Merged image showing the colocalization of LCN2 with 7/4 in yellow. The shaded area in the inset indicates the infarcted region. (**I**) The percentage of LCN2-positive cell types (*n* = 5). Scale bars, 10 μm (**A**–**D**), 50 μm (**E**–**H**).

The BBB is a gliovascular unit composed of cerebral endothelial cells and pericytes surrounded by astrocytic end-feet [22,23]. Infiltration of neutrophils following ischemic stroke contributes to the disruption of the BBB. In order to examine if LCN2 was induced in the components of the BBB in the current study, mice were subjected to 1 h of tMCAo followed by 23 h of reperfusion. Brain sections were stained with DyLight594-labeled tomato lectin (blood vessel marker), anti-GFAP (astrocyte), and anti-LCN2 antibodies (Figure 1). Tomato lectin is a glycoprotein with specific affinity for the plasma membrane and cytoplasm of vascular endothelial cells [24]. Although LCN2 was absent in non-ischemic brain tissues (Figure 1A–C), LCN2 was detected as a thin layer on the inner surface of vascular endothelial cells at 23 h after tMCAo (Figure 1D–L). The LCN2-positive blood vessel is probably the pial artery penetrating from the brain surface [25]. LCN2 was also detected in the astrocytes with end-feet surrounding the blood vessels (Figure 2A–D), as well as infiltrating neutrophils stained positively with a specific marker for neutrophils (7/4) (Figure 2E–H). The numbers and percentages of LCN2-positive cells in the ipsilateral hemisphere at 23 h of reperfusion were counted and calculated: LCN2-positive blood vessels (135 ± 29 cells/mm^2^, 29.2 ± 2.3%), neutrophils (287 ± 61 cells/mm^2^, 60.7 ± 2.2%), and astrocytes (59 ± 27 cells/mm^2^, 10.1 ± 4.4%) (Figure 2I).

### 2.2. LCN2 Monoclonal Antibody (mAb) Specifically Targeted Recombinant and Endogenous LCN2 Proteins

**Figure 3:**
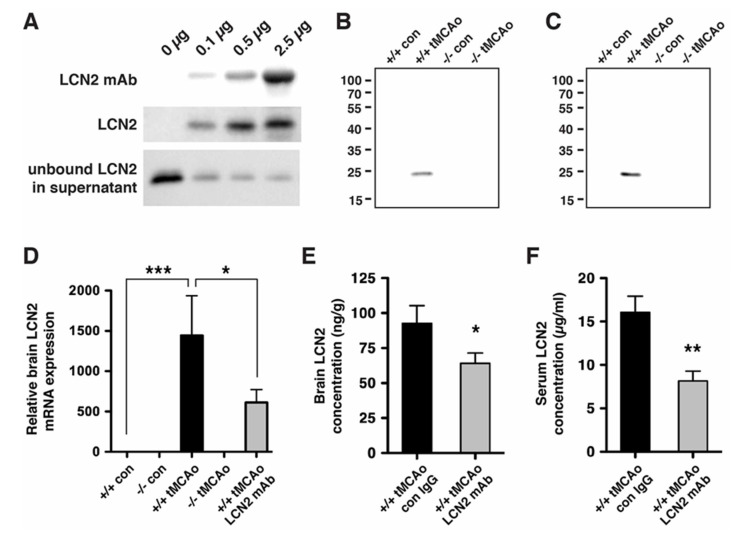
LCN2 monoclonal antibody (mAb) specifically immunoprecipitated recombinant and endogenous LCN2 proteins. (**A**) Representative Western blots showing that LCN2 mAb reduced the level of LCN2 protein by immunoprecipitation. Increasing concentrations of LCN2 mAb (0, 0.1, 0.5, and 2.5 μg) bound to the Dynabeads were incubated with a fixed amount of mouse recombinant LCN2 protein (0.1 μg). LCN2 mAb bound to the Dynabeads, immunoprecipitated LCN2 protein, and unbound LCN2 protein in the supernatant after the immunoprecipitation are shown in the top, middle, and bottom panels, respectively; (**B**,**C**) LCN2 mAb specifically immunoprecipitated the LCN2 protein that was induced after tMCAo. Ipsilateral hemisphere lysates (**B**) and blood sera (**C**) collected from naive *LCN2^+/+^* and *LCN2^−/−^* mice (+/+ con and −/− con) and at 23 h after tMCAo (+/+ tMCAo and −/− tMCAo) were immunoprecipitated with LCN2 mAb and analyzed by Western blotting using a polyclonal antibody that recognized LCN2 protein; (**D**) Total RNA isolated from ipsilateral hemispheres of naive *LCN2^+/+^* and *LCN2^−/−^* mice (+/+ con and −/− con), at 23 h after tMCAo (+/+ tMCAo and −/− tMCAo), and *LCN2^+/+^* mice treated with LCN2 mAb at 4 h after tMCAo (+/+ tMCAo LCN2 mAb) was analyzed by real-time RT-PCR (*n* = 6 per group). Relative mRNA expression of LCN2 in the brain homogenates was compared between the mice groups using a one-way ANOVA and Newman–Keuls post hoc tests. LCN2 mRNA levels were significantly induced after tMCAo (*** *p* < 0.001) as compared with those in naive *LCN2^+/+^* mice. LCN2 mRNA levels in mice that were treated with LCN2 mAb were significantly reduced (* *p* < 0.05) as compared those in *LCN2^+/+^* mice after tMCAo; (**E**,**F**) Mice were treated with an isotype control IgG (con) or LCN2 mAb at 4 h after tMCAo. We analyzed the concentration of LCN2 in the ipsilateral hemispheres (*n* = 5 per group, **E**) and blood sera (*n* = 9–10 per group, **F**) at 23 h after reperfusion using ELISA. The concentration of LCN2 in the brains of mice treated with LCN2 mAb were significantly decreased (* *p* < 0.05) as compared with that in the brains of mice that received the control IgG (one-tailed, unpaired *t* test). The serum concentration of LCN2 in mice that received LCN2 mAb were also significantly decreased (** *p* < 0.01) as compared with that in mice that received the control IgG (two-tailed, unpaired *t*-test).

LCN2 was induced after tMCAo (Figure 1 and Figure 2) and secreted into the extracellular space (e.g., blood) where it may be accessible to therapeutic antibodies [19]. To assess whether LCN2 mAb would bind to the LCN2 protein in solution, we performed immunoprecipitation with recombinant LCN2 protein and found that LCN2 mAb immunoprecipitated recombinant LCN2 protein in a dose-dependent manner (Figure 3A). To assess whether LCN2 mAb targeted endogenous LCN2 that was induced after stroke, brain homogenates and blood sera of *LCN2^+/+^* and *LCN2^−/−^* mice collected before and after tMCAo were analyzed by immunoprecipitation. A single protein band of LCN2 at approximately 25 kD was immunoprecipitated with LCN2 mAb in brain homogenates (Figure 3B) and blood sera (Figure 3C) from *LCN2^+/+^* mice after tMCAo, but not *LCN2^−/−^* mice.

LCN2 is an autocrine mediator of reactive astrocytosis [26]; therefore, the level of secreted LCN2 may play a role in the autoregulatory mechanism. Since LCN2 induction peaked at 23 h after tMCAo [19], LCN2 mAb was administered at 4 h after tMCAo to assess the effects of LCN2 mAb in vivo. Treatment with LCN2 mAb significantly attenuated the induction of LCN2 mRNA (Figure 3D) and the levels of LCN2 proteins in the ipsilateral hemispheres (Figure 3E) and blood sera (Figure 3F) at 23 h after tMCAo.

### 2.3. Neurological Deficits and Cerebral Infarction after tMCAo were Reduced after Treatment with LCN2 mAb

**Figure 4:**
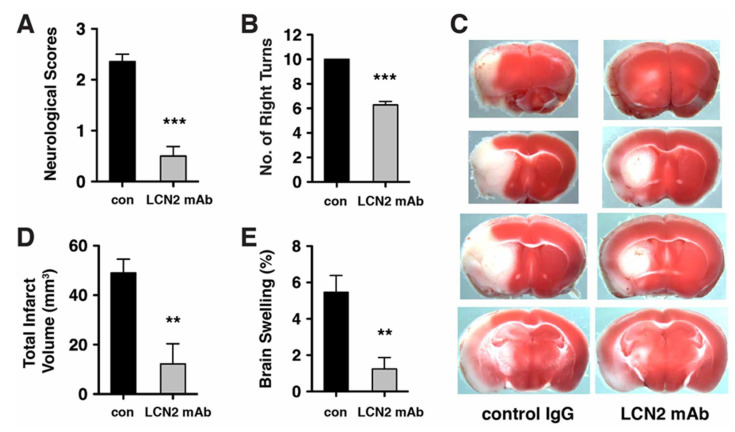
LCN2 mAb attenuated neurological deficits and cerebral infarction after tMCAo. Neurological deficit scoring (**A**) and corner test (**B**) were performed at 20 h after one hour of tMCAo in mice treated with isotype control IgG (con) and LCN2 mAb (*n* = 7 per group). (**C**) Representative images of TTC-stained brain slices from mice treated with control IgG and LCN2 mAb after 23 h of reperfusion. Viable tissue is stained in red color, whereas the infarcted area remains unstained (white). Total infarct volume (**D**) and brain swelling percentage (**E**) in mice treated with LCN2 mAb were significantly decreased 23 h after reperfusion as compared with those in mice treated with the control IgG (*n* = 5 per group). ** *p* < 0.01, *** *p* < 0.001 compared with treatments with control IgG (two-tailed, unpaired *t*-test).

The therapeutic effects of LCN2 mAb were assessed by administering LCN2 mAb before the complete rise of LCN2. Treatment with LCN2 mAb at 4 h after tMCAo significantly reduced stroke induced neurological deficits (Figure 4A) and sensorimotor asymmetry (Figure 4B) as compared with isotype control antibody treatment. Improvement in neurological outcome after LCN2 mAb treatment was reflected in cerebral infarction and edema (Figure 4C–E). Total infarct volume in mice treated with LCN2 mAb was reduced by ~75% compared with that in mice treated with the control antibody (49.01 ± 5.58 mm^3^ for control antibody versus 12.25 ± 8.11 mm^3^ for LCN2 mAb; Figure 4D). Brain swelling in the ipsilateral hemispheres of mice treated with LCN2 mAb was reduced by ~77% compared with that in the ipsilateral hemispheres of mice treated with the control antibody (5.47% ± 0.91 for control antibody versus 1.24% ± 0.63 for LCN2 mAb; Figure 4E).

### 2.4. Blood–Brain Barrier Breakdown and Infiltration of Neutrophils after tMCAo were Reduced after Treatment with LCN2 mAb

**Figure 5:**
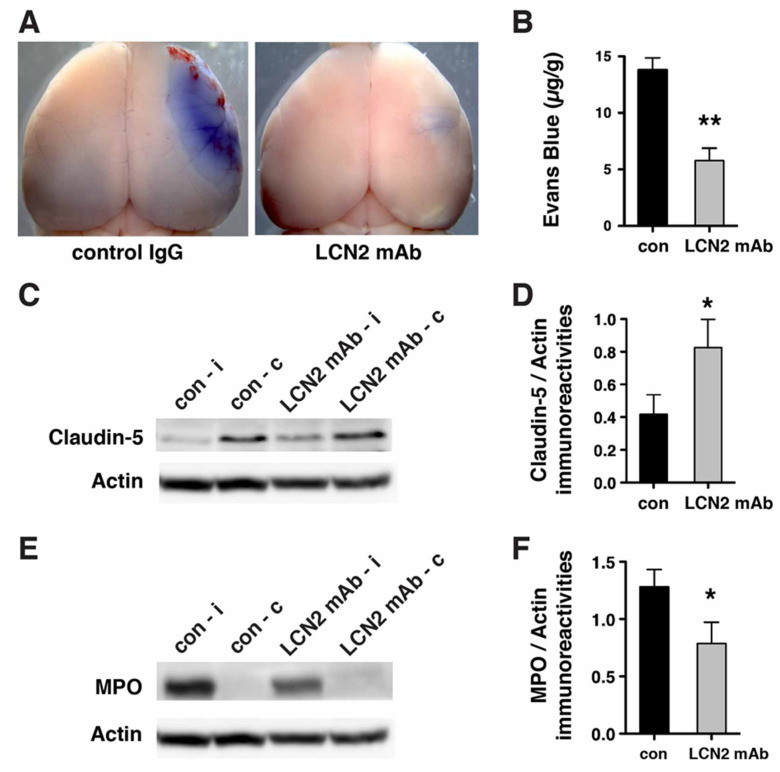
LCN2 mAb limited blood–brain barrier leakage and infiltration of neutrophils after tMCAo. Representative images (**A**) and quantification (**B**) of Evans blue extravasation in the ipsilateral hemispheres of mice treated with control IgG (con) and LCN2 mAb (*n* = 5 per group) after one hour of tMCAo and 23 h after reperfusion. The concentration of Evans blue dye in the ipsilateral hemispheres of mice treated with LCN2 mAb was significantly decreased (** *p* < 0.01) as compared with that in the ipsilateral hemispheres of mice treated with control IgG (two-tailed, unpaired *t* test); (**C**) The expression level of the tight junction protein claudin-5 was analyzed after treatments with control IgG and LCN2 mAb (*n* = 4 per group). The ipsilateral (i) and contralateral (c) hemispheres isolated at 23 h after tMCAo were analyzed by Western blotting using antibodies against claudin-5. Representative Western blot showing the expression of claudin-5 (~22 kDa) in brain homogenates. β-actin served as a loading control; (**D**) The level of claudin-5 immunoreactivity normalized to β-actin (claudin-5/actin) in the ipsilateral hemispheres in mice treated with LCN2 mAb was significantly higher than that in the ipsilateral hemispheres of mice that received the control IgG (* *p* < 0.05, one-tailed, unpaired *t* test); (**E**,**F**) Neutrophil infiltration was analyzed by measuring the levels of MPO in brain homogenates. The ipsilateral (i) and contralateral (c) hemispheres of mice treated with control IgG (con) and LCN2 mAb (*n* = 4 per group) isolated at 23 h after tMCAo were analyzed by Western blotting using antibodies against MPO; (**E**) Representative Western blots show the expression of MPO heavy chain (~55 kD) in brain homogenates; (**F**) The level of MPO immunoreactivity normalized to β-actin (MPO/actin) was significantly reduced in the ipsilateral hemispheres of mice treated with LCN2 mAb (* *p* < 0.05, one-tailed, unpaired *t* test) as compared with that in the ipsilateral hemisphere of mice that received control IgG.

Since LCN2 was induced in cerebral endothelial cells and astrocytes gatekeeping the BBB after tMCAo (Figure 1 and Figure 2), we assessed the effectiveness of LCN2 mAb to treat the BBB disruption after stroke (Figure 5). Evans blue extravasation assays showed that LCN2 mAb attenuated the leakage of BBB after tMCAo (Figure 5A). The amount of Evans blue dye extravasated in the ipsilateral hemispheres of mice treated with LCN2 mAb was reduced by ~59% as compared with mice treated with the control antibody (13.79 ± 1.08 µg/g for control antibody versus 5.78 ± 1.10 µg/g for LCN2 mAb; Figure 5B). Claudin-5, a tight junction protein that regulates the permeability of the BBB, was deteriorated in the ipsilateral hemispheres after tMCAo (Figure 5C) [6]. Treatment with LCN2 mAb attenuated the deterioration of claudin-5 after tMCAo (Figure 5D).

Previous studies have reported that infiltration of peripheral neutrophils during reperfusion exacerbates BBB disruption by releasing ROS and proteases [6,9]. Therefore, we assessed the infiltration of neutrophils by Western blotting, using an antibody that recognized myeloperoxidase (MPO), which is a key enzyme that is expressed in blood-borne neutrophils [27]. Although the MPO-positive neutrophils were accumulated in the ipsilateral hemisphere 23 h after tMCAo, they were absent in the contralateral hemisphere (Figure 5E). The level of MPO was significantly reduced after LCN2 mAb treatment as compared with mice treated with the control antibody (Figure 5F). These findings suggest that treatment with LCN2 mAb may reduce neutrophil infiltration after tMCAo.

**Figure 6:**
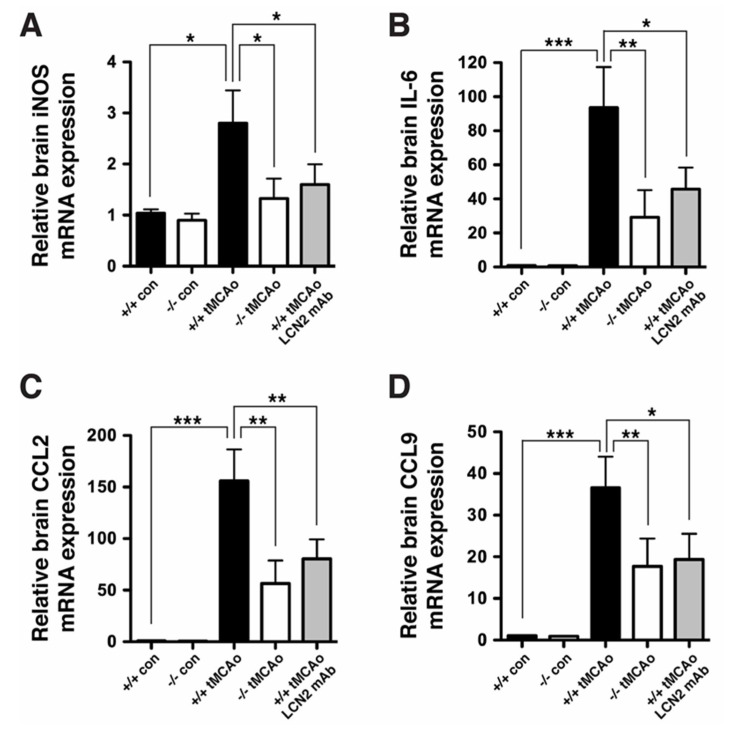
LCN2 mAb attenuated the induction of chemokines and cytokines after tMCAo. We analyzed total RNA isolated from ipsilateral hemispheres of naive *LCN2^+/+^* and *LCN2^−/−^* mice (+/+ con and −/− con), as well as at 23 h after tMCAo (+/+ tMCAo and −/− tMCAo), and *LCN2^+/+^* mice treated with LCN2 mAb at 4 h after tMCAo (+/+ tMCAo LCN2 mAb) using real-time RT-PCR (*n* = 6 per group). We compared differences in the relative mRNA expression of iNOS (**A**), IL-6 (**B**), CCL2 (**C**), and CCL9 (**D**) between the five groups using a one-way ANOVA and Newman–Keuls post hoc tests. The relative brain mRNA levels of iNOS, IL-6, CCL2, and CCL9 after tMCAo were significantly increased as compared with those in naive *LCN2^+/+^* mice. Additionally, the mRNA levels of iNOS, IL-6, CCL2, and CCL9 in *LCN2^−/−^* mice or mice treated with LCN2 mAb were significantly reduced as compared with those in *LCN2^+/+^* mice after tMCAo (*** *p* < 0.001, ** *p* < 0.01, * *p* < 0.05).

Pro-inflammatory ROS, cytokines, and chemokines are induced after tMCAo to recruit neutrophils to ischemic brain tissues [9], and LCN2 has been identified as a chemokine inducer in the CNS [28]. Real-time RT-PCR analysis revealed that inducible nitric oxide (NO) synthase (iNOS), an enzyme that is responsible for producing NO; pro-inflammatory cytokines (IL-6); and chemokines (CCL2, CCL9) were significantly induced after tMCAo (Figure 6). The induction of iNOS, IL-6, CCL2, and CCL9 was significantly attenuated in LCN2 deficient mice and mice treated with LCN2 mAb at 4 h after tMCAo. These findings suggest that LCN2 promotes neuroinflammation and LCN2 mAb attenuates neuroinflammation after ischemic stroke (Figure 6).

### 2.5. Epitope Mapping of LCN2 mAb

**Figure 7:**
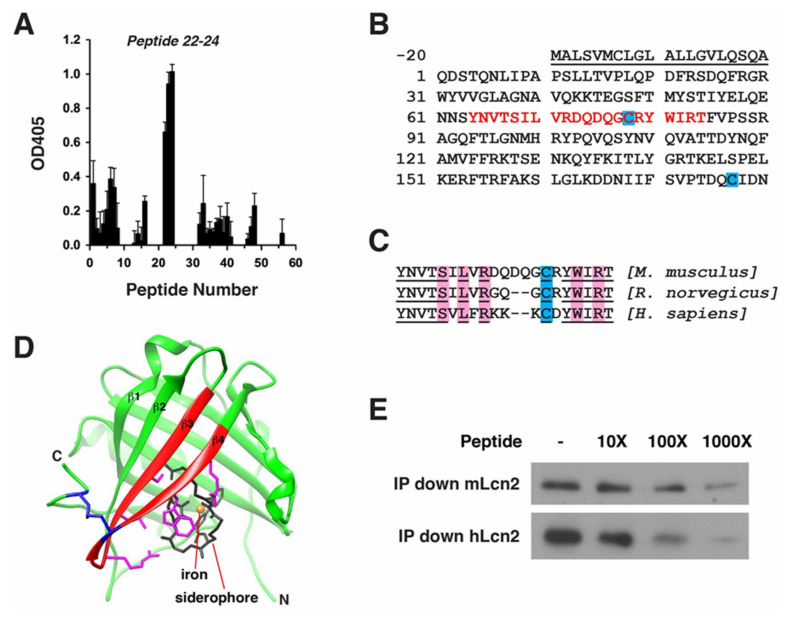
Epitope mapping of LCN2 mAb. (**A**) ELISA quantification of relative binding of LCN2 mAb to 56 peptides covering the mouse LCN2 protein (*n* = 3); (**B**) The combined sequences of peptides 22–24 are shown in red at amino acids 64–84 of the mouse LCN2 protein. The N-terminal signal peptide that was cleaved prior to the secretion of the protein is underlined and not included in the epitope mapping. Cys-78 and Cys-177 forming an intramolecular disulfide bond (blue); (**C**) Alignment of the LCN2 mAb epitope sequences from mouse, rat, and human LCN2 homologs. Conserved amino acids are underlined. Amino acids interacting with siderophore are highlighted in pink. Conserved cysteines are highlighted in blue; (**D**) The crystal structure of human LCN2 protein containing a siderophore (black) and an iron (orange). The epitope of LCN2 mAb, highlighted in red, is located in the β3 and β4 strands. The side chains of amino acids bound with siderophore are colored in pink. An intramolecular disulfide bond formed between Cys-78 in the epitope and Cys-177 near the C-terminal (blue); (**E**) Representative Western blot showing that increased concentrations of epitope peptides from mouse (YNVTSILVRDQDQGCRYWIRT) and human (YNVTSVLFRKKKCDYWIRT) tissue reduced the level of immunoprecipitated mouse (mLCN2) or human LCN2 protein (hLCN2).

In light of the therapeutic benefits of LCN2 mAb after tMCAo, the binding epitope of LCN2 mAb was mapped to reveal the molecular effects of LCN2 mAb (Figure 7). Using an ELISA with a library of fifty-six 15-mer peptides covering mouse LCN2, we discovered that LCN2 mAb demonstrated a higher affinity for peptides 22–24 (Figure 7A). The sequences spanning peptides 22–24 were identified as amino acids 64–84 of the mouse LCN2 protein (Figure 7B). The residues within the epitope are highly conserved between mouse, rat, and human LCN2 homologs (Figure 7C), suggesting that the LCN2 mAb may also interact with rat and human LCN2 proteins, and that the binding epitope is functionally conserved as well. A ribbon model of mouse LCN2 crystal structure (PDB: 1 × 89) was generated using UCSF Chimera (http://www.cgl.ucsf.edu/chimera), and predicted that the LCN2 mAb epitope is located in the solvent-accessible β3 and β4 strands (Figure 7D) [29,30]. To confirm the epitope mapping, mouse and human epitope peptides were included in immunoprecipitation analyses of recombinant mouse and human LCN2 proteins (Figure 7E). LCN2 mAb immunoprecipitated both mouse and human LCN2 proteins. Moreover, LCN2 epitope peptides competed with LCN2 proteins for LCN2 mAb, reducing the level of immunoprecipitated LCN2 proteins in a dose-dependent manner.

## 3. Discussion

Thrombolysis has been widely used to treat acute ischemic stroke, but reperfusion may initiate oxidative stress and inflammatory responses and worsen neurological outcomes [2]. We believe that this study is the first to validate the concept that neutralization of LCN2 is a plausible therapeutic strategy to reduce stroke-reperfusion injury. Our findings provide clear evidence that LCN2 mRNA and protein are absent in the brain under non-ischemic conditions. We observed an acute induction of LCN2 protein after stroke in infiltrating neutrophils, cerebral endothelial cells, and astrocytes gatekeeping the BBB. LCN2 mAb specifically immunoprecipitated recombinant LCN2 protein, as well as endogenous LCN2 protein induced in blood sera and ischemic brain tissues after stroke. Treatments with LCN2 mAb within a clinically relevant time window significantly attenuated the induction of LCN2 mRNA and protein, pro-inflammatory mediators (iNOS, IL-6, CCL2, CCL9), infiltration of neutrophils, BBB leakage, and cerebral infarction, and improved functional outcomes after stroke.

The members of the lipocalin family generally serve as transporters that carry small hydrophobic molecules through a funnel-like binding pocket. The LCN2 crystal structure consists of an eight-stranded antiparallel β-barrel that is clenched together by an α-helix and a disulfide bond formed between Cys-78 and Cys-177, which are located on opposite sides of the molecule (Figure 7) [29]. The cup-shaped pocket of LCN2 enclosed within the β-pleated sheets carries an iron-loaded siderophore. Identification of the antigenic epitopes is a critical step in understanding the specificity and molecular effects of mAb [31]. The LCN2 mAb epitope was mapped to β3 and β4 strands, containing the residues that are responsible for forming the intramolecular disulfide bond (Cys-78) and binding with siderophore (Ser-68, Leu-70, Arg-72, Trp-81, Arg-83); therefore, binding with LCN2 mAb may disrupt the formation of the disulfide bond, destabilize the cup-shaped structure of LCN2, and interfere with iron transportation (Figure 7C,D). The mapped epitope of LCN2 mAb may also serve as a foundation for additional development of neutralizing antibodies and their derivatives for therapeutic and diagnostic purposes.

The initial injuries caused by cerebral ischemia are acute and cannot usually be mended [32]. Stroke-induced oxidative stress and neuroinflammation evolve over a period of hours to days, or even months. The protracted periods of oxidative stress and neuroinflammation provide a window of opportunity for therapeutic interventions. Immunotherapy designed to target pro-inflammatory mediators as a means of improving stroke outcome has, therefore, attracted considerable scientific attention [20,21]. Neuroprotective effects of humanized antibodies against the NMDA receptor, intercellular adhesion molecule-1 (ICAM-1), and E-selectin have been demonstrated in pre-clinical stroke models. Each of these antibody-based therapies, however, failed in clinical trials due to severe side effects including psychotomimetic symptoms, fever, pneumonia, pulmonary edema, and cardiac arrest. Since LCN2 is not expressed in the brain under non-ischemic conditions, it is possible that unwanted side effects would be minimal following treatments with LCN2 mAb.

Disruption of the BBB and recruitment of peripheral immune cells into ischemic brain tissues are the major components of neuroinflammation. Neutrophils are attracted by the local release of ROS, cytokines, and chemokines to adhere and migrate through the endothelium of the cerebral microvasculature [9]. LCN2 was induced on the inner surface of cerebral endothelial cells and astrocytes whose end-feet encircle blood vessels (Figure 1 and Figure 2), suggesting that LCN2 induction sites are in direct contact with infiltrating neutrophils to accelerate their transmigration. Previous studies using LCN2-deficient mice have identified LCN2 as a chemokine inducer in the CNS in an autocrine or paracrine manner [11,18,28]. Our results, using pharmacological approaches, are in line with previous genetic studies. LCN2 mAb administered before the complete rise of LCN2 after stroke not only attenuated the levels of LCN2 itself, but also reduced the induction of pro-inflammatory chemokines and cytokines.

The pro-inflammatory mediators regulated by LCN2 have been demonstrated to play key roles in stroke-reperfusion injury [32]. Large amounts of nitric oxide (NO) are produced after stroke by iNOS, which is mainly expressed in microglia, astrocytes, endothelial cells, and infiltrating neutrophils [32,33]. NO reacts preferentially with ROS and forms peroxynitrite anions (ONNO^−^), which are cytotoxic to mitochondrial enzymes and genetic materials. Inhibition of iNOS production in infiltrating neutrophils and cerebral endothelial cells provides extended neuroprotection after transient and permanent cerebral ischemia [34]. IL-6 is a pro-inflammatory cytokine that is elevated in blood plasma and the brain at 3–24 h following experimental stroke [35]. Findings from a previous study revealed that exposure to IL-6 in vitro disrupts the integrity of the BBB by decreasing the transendothelial electrical resistance (TEER) of rat cerebral endothelial cells [36]. CCL2 (monocyte chemoattractant protein-1, MCP-1) and CCL9 (macrophage inflammatory peptide gamma, MIP-1γ) are chemokines upregulated after ischemic stroke in humans [37] and rodents [38]. Additionally, CCL2 and its receptor CCR2 have been implicated in post-stroke leukocyte trafficking [39]. Further, studies have demonstrated that the genetic deletion of CCL2 [40] and CCR2 [41] reduced the permeability of the BBB, the accumulation of immune cells in ischemic brain tissues, and subsequent cerebral infarction [42]. The role of CCL9 has not been investigated in stroke, but deficiency of its receptor, CCR1, attenuated neutrophil adherence to vascular endothelium and transmigration to post-ischemic tissues [43]. Taken together, these studies show that genetic or pharmacological inhibition of these pro-inflammatory mediators (iNOS, IL-6, CCL2, CCL9) provides neuroprotection against stroke. Nevertheless, targeting LCN2 to blunt post-stroke neuroinflammation may be more advantageous than inhibiting individual cytokines and chemokines because LCN2 is likely an important upstream regulator of these mediators in the inflammatory cascade.

The LCN2 mAb may have wide applicability in multiple CNS disorders. Preclinical studies showed that LCN2 deficiency markedly decreased neuroinflammation and associated injuries in mouse models of traumatic brain injury [44,45], hemorrhagic stroke [46,47,48], spinal cord injury [49], and experimental autoimmune encephalomyelitis [50]. These findings suggest that LCN2 is a critical mediator of neuroinflammation in response to various CNS disorders. Neutralization of LCN2 using LCN2 mAb may inhibit or reverse neuroinflammatory responses in these CNS disorders [11]. Although neutralizing the activity of extracellular LCN2 protein by LCN2 mAb is validated in this study, other therapeutic approaches that inhibit the expression and secretion of LCN2 [51] or interfere with the interaction between LCN2 and its receptors [52] are also potential avenues for therapeutic development [11].

## 4. Materials and Methods

### 4.1. Ischemic Stroke Model and LCN2 mAb Treatment

Male *LCN2^+/+^* and *LCN2^−/−^* mice on a C57BL/6 background (Jackson Laboratory, Bar Harbor, ME, USA) between 2 and 4 months of age were used for experiments. Only male mice were used in this study because of the sexual dimorphism in ischemic stroke [53]. Female mice are protected after tMCAo in pro-estrus and estrus phases when estrogen is relatively high [54]. Because of the higher mortality and poorer functional outcome in aged mice after tMCAo [54], young males were used to minimize the cost of LCN2 mAb and raising animals. Focal cerebral ischemia was induced by transient middle cerebral artery occlusion (tMCAo) for 1 h with a silicon-coated monofilament suture (Cat # 602312PK10Re, Doccol, Sharon, MA, USA) [8,19,55]. Mice were injected intraperitoneally (i.p.) with 100 µg of LCN2 mAb (~4 mg/kg, Cat# MAB18571, R&D Systems, Minneapolis, MN, USA) or isotype control antibody (~4 mg/kg, Cat# MAB006, R&D) at 4 h after tMCAo. Regional cerebral blood flow (rCBF) was continuously monitored by laser Doppler flowmetry (Perimed, Ardmore, PA, USA) during tMCAo [8,19]. Mice were excluded from final analysis if sufficient occlusion (<30% of the baseline) and reperfusion (>80% of the baseline) was not attained, if excessive bleeding occurred during surgery, or if hemorrhage was found in the brain slices or at the base of the circle of Willis during post-mortem examination. A total of 108 mice were used in the study (Table 1). Mortality rate after tMCAo was similar between the groups.

All procedures were approved by the Institutional Animal Care and Use Committee at Kent State University and National Health Research Institutes, and were in accordance with National Institutes of Health and ARRIVE (Animal Research: Reporting In Vivo Experiments) guidelines.

### 4.2. Immunofluorescence Staining

Mice were anesthetized with 4% isoflurane in N_2_O/O_2_ (70%/30%) at 23 h after tMCAo and perfused transcardially with 4% PFA (Cat# P6148, Sigma-Aldrich) in 0.15 M phosphate buffer (pH 7.3) [19,56]. Brains were rapidly dissected and sectioned using a brain matrix (Cat# BS-2000C, Braintree Scientific, Braintree, MA, USA) to generate 1-mm-thick coronal sections for paraffin embedding. Immunohistochemistry (IHC) antibody diluent buffer containing 1× PBS, 0.1% Triton X-100, 3% BSA, and 2% normal donkey serum (Cat# 017-000-121, Jackson ImmunoResearch, West Grove, PA, USA) was prepared. Paraffin sections (5 μm) containing striatum (~0.49 mm to bregma) were fixed with 4% PFA and incubated overnight at 4 °C with goat anti-LCN2 (Cat# AF1857, R&D; 1:200 dilution in IHC antibody diluent buffer), rabbit anti-GFAP (Cat# Z0334, DAKO, Glostrup, Denmark; 1:1000 dilution in IHC antibody diluent buffer), and rat anti-Ly-6B.2 clone 7/4 (Cat# ab53457, Abcam; 1:200 dilution in IHC antibody diluent buffer). After washing, the sections were stained with Alexa Fluor conjugated secondary antibodies (Cat# A-11055, Thermo Fisher Scientific, Waltham, MA, USA; Cat# 711-585-152, Cat# 712-585-153, Jackson ImmunoResearch; 1:200 dilution in IHC antibody diluent buffer), DyLight649-labeled tomato lectin (Cat# DL-1178, Vector Laboratories, Burlingame, CA, USA; 1:200 dilution in IHC antibody diluent buffer), and mounted in media containing 4′, 6-diamidino-2-phenylindole (DAPI) (Cat# H-1200, Vector Laboratories, Burlingame, CA, USA). The images were acquired using a Leica TCS SP5 II confocal laser scanning microscope. The number of LCN2-positive cells were counted by an investigator blinded to the treatments in six separate fields (400 μm × 400 μm) within the ipsilateral hemisphere using Leica Application Suite X (LAS X) software. The numbers of cells per mm^2^ were statistically analyzed between different groups.

### 4.3. Immunoprecipitation and Western Blot Analysis of Recombinant LCN2 Protein

Dynabeads Protein G (50 µL, Cat# 10007D, Thermo) was washed three times with 1X PBS containing 0.02% Tween-20 (PBST) [57]. After washing, the Dynabeads were incubated with different concentrations (0, 0.1, 0.5 and 2.5 μg) of LCN2 mAb (Cat# MAB18571, R&D) in 100 µL PBST for 30 min at room temperature. LCN2 mAb bound on the Dynabeads was incubated with 0.1 μg of recombinant mouse LCN2 protein (Cat# 1857-LC, R&D) in 100 µL PBST for 1 h at 4 °C. LCN2 protein-LCN2 mAb complex on the Dynabeads and unbound LCN2 protein in the supernatant were separated by 10% SDS-PAGE, transferred to PVDF membrane, and analyzed by Western blotting. LCN2 mAb bound on the Dynabeads was detected by HRP goat anti-Rat IgG2A (Cat# PA1-84709, Thermo; 1:1000 dilution in Western Antibody Dilution Buffer containing 1× TBS, 0.1% Tween, and 2% nonfat dry milk). The immunoprecipitated LCN2 protein and unbound LCN2 protein in supernatant were detected using goat anti-LCN2 antibody (Cat# AF1857, R&D; 1:1000 dilution in Western Antibody Dilution Buffer) and EasyBlot anti-goat IgG kit (Cat# GTX228910-01, GeneTex, Hsinchu City, Taiwan). Immunoreactive bands were detected using SuperSignal West Pico chemiluminescence substrate (Cat# 34080, Thermo) and imaged using a chemiluminescence imager Celvin S420 and SnapAndGo version1.8 (Biostep Gmbh, Burkhardtsdorf, Germany).

### 4.4. Immunoprecipitation, Western Blot, and ELISA Analysis of Endogenous LCN2 Protein in Mouse Brain and Blood Serum

Mice were anesthetized with 5% isoflurane and sacrificed by cervical dislocation at 23 h after tMCAo [8,19,57]. Ipsilateral hemisphere was isolated and homogenized using 15 strokes of a Teflon-glass homogenizer in ice-cold RIPA buffer containing 1X Halt Protease and Phosphatase inhibitor, 5 mM EDTA, 10 nM Pepstain A, and 1 mM PMSF (Cat# 78442, Thermo). The homogenate was passed through a 25G 5/8 needle five times and centrifuged at 10,000× *g* for 10 min at 4 °C. The supernatant was collected for immunoprecipitation, Western blotting, and ELISA.

Mouse blood was collected from decapitated trunk and placed at room temperature for 0.5 h [19]. The blood was centrifuged at 2000× *g* for 10 min at 4 °C, and the supernatant was collected as blood serum for immunoprecipitation, Western blotting, and ELISA.

LCN2 mAb (Cat# MAB18571, R&D) was crosslinked to the Dynabeads (Cat# 10007D, Thermo) using BS3 bis(sulfosuccinimidyl)suberate (Cat# A39266, Thermo) following the manufacturer’s protocol [57]. Brain homogenate and serum was incubated overnight at 4 °C with LCN2 mAb–Dynabeads. The immunoprecipitated proteins were separated by 10% SDS-PAGE, transferred to PVDF membrane, and analyzed by Western blotting using goat anti-LCN2 antibody (Cat# AF1857, R&D; 1:1000 dilution in Western Antibody Dilution buffer) and EasyBlot anti-goat IgG kit (Cat# GTX228910-01, GeneTex).

The level of LCN2 protein in brain homogenate and blood serum was quantified following the manufacturer’s protocols for mouse lipocalin-2/NGAL Quantikine ELISA kit (Cat# MLCN20, R&D) [19].

### 4.5. Real-Time RT-PCR

Total RNA was extracted from the ipsilateral (right) hemisphere using 1 mL of TRIzol reagent (Cat# 15596-018, Invitrogen, Carlsbad, CA, USA) and MagNA Lyser Green Beads (Cat# 03 358 941 001, Roche) at 5000 rpm for 20 s in a MagNA Lyser Instrument (Roche) [18]. After 20 s, the lysates were cooled down on ice for 10 s to prevent RNA degradation and extracted again at 5000 rpm for 20 s. The lysates were centrifuged at 12,000× *g* for 5 min at 4 °C. The supernatant was mixed well with 0.2 mL of 1-Bromo-3-chloropropane (Cat# B9673, Sigma-Aldrich) by shaking vigorously for 20 s and set at room temperature for 3 min. The mixtures were centrifuged at 12,000× *g* at 4 °C for 15 min. The upper aqueous phase was transferred to a new Eppendorf tube after centrifugation. The RNA in the aqueous phase was precipitated by 0.5 mL of isopropanol (Cat# I9516, Sigma-Aldrich). Isolated RNA was quantified with a NanoDrop 2000 Spectrophotometer (Thermo) and reverse transcribed into cDNA using a RevertAid H Minus First Strand cDNA Synthesis Kit (CAT# K1632, Thermo) in a Veriti 96-Well Thermal Cycler (Thermo). Real-time PCR was conducted using the Luminaris Color HiGreen High ROX qPCR Master Mix (CAT# K0363, Thermo) and the StepOnePlus™ Real-Time PCR System (CAT# 4376600, Thermo). The threshold cycle (*C*_t_) values for all qPCR results are shown in Supplementary Figure S1. The mRNA levels of LCN2, iNOS, IL-6, CCL2, and CCL9 were analyzed by the 2^−∆∆Ct^ method, using glyceraldehyde-3-phosphate dehydrogenase (GAPDH) mRNA as an internal control. The sequences of the primers used are listed in Table 2 [18,26]. We used GAPDH mRNA as the reference gene to obtain Δ*C*_t_ (Formula (1)), and used the expression level of target gene in naive *LCN2^+/+^* mice as the reference sample to obtain ΔΔ*C*_t_ (Formula (2)) [58]. The following are the formulas.

Formula (1): Δ*C*_t_ = *C*_t_ (a target gene) − *C*_t_ (a reference gene)

Formula (2): ΔΔ*C*_t_ = Δ*C*_t_ (a target sample) − Δ*C*_t_ (a reference sample).

### 4.6. Neurological Deficits and Corner Tests

Since somatosensory asymmetry occurs frequently in stroke patients with right cerebral hemisphere damage [59,60] and MCA occlusion typically results in extensive neuronal death in striatum and cortex, sensorimotor and postural asymmetries were examined using neurological deficit scores and corner tests. Mice were assessed for neurological deficits at 23 h after tMCAo using a four-tiered grading system [8,19,55]. Score 0, no observed neurological deficit (normal); score 1, inability to walk straight (mild); score 2, circling toward the paretic side (moderate); score 3, falling on the paretic side (moderate–severe); score 4, loss of the righting reflex (severe). For the corner test, mice were placed between two vertical boards (30 × 18 × 0.7 cm^3^) attached at a 30° angle in the home cage [19,61]. The numbers of right (ipsilateral) and left turns when the mice reached the wedge of the corner were recorded. Ten trials were performed for each mouse.

### 4.7. Determination of Infarct Volume and Brain Swelling

Mice were deeply anesthetized with 4% isoflurane and euthanized by cervical dislocation at 23 h after tMCAo. Mouse brains were sliced coronally at 1-mm intervals and stained with 2% 2,3,5-triphenyltetrazolium chloride (TTC; CAT# T8877, Sigma-Aldrich, St. Louis, MO, USA) in PBS at room temperature for 20 min [8,19,55]. Front and rear sides of brain slices were photographed using a Leica EZ4HD stereomicroscope with an integrated high definition digital camera. Areas of cerebral infarction, ipsilateral, and contralateral hemispheres were assessed using the NIH ImageJ by an investigator blinded to treatment allocation. Infarct volumes, brain swelling, and shrinkage were calculated from the following equations. Infarct area on one side of brain section (mm^2^) = area of contralateral hemisphere – (area of ipsilateral hemisphere – area of infarct area). Total infarct volume (mm^3^) = (front infarct area of a section + rear infarct area of the same section)/2 × thickness of the section × total numbers of sections. Brain swelling (%) = ((ipsilateral hemisphere volume – contralateral hemisphere volume)/contralateral hemisphere volume) × 100. Brain shrinkage (%) = ((contralateral hemisphere volume–ipsilateral hemisphere volume)/contralateral hemisphere volume) × 100.

### 4.8. Determination of Blood–Brain Barrier Leakage

A 2% Evans blue (Cat# E2129, Sigma-Aldrich) dye in sterilized saline was injected into the right jugular vein (3 mL/kg of body weight) immediately after tMCAo [62]. Mice were perfused intracardially with 50 mL saline at 23 h after tMCAo. Ipsilateral hemisphere was weighted, homogenized in 1 mL PBS, and centrifuged at 15,000 rpm for 30 min at 4 °C. The supernatant was mixed with the same volume of 50% trichloroacetic acid (Cat# T6399, Sigma-Aldrich), incubated at 4 °C overnight, and centrifuged at 15,000 rpm for 30 min at 4 °C. The absorbance of supernatant was measured at 610 nm using a spectrophotometer to determine Evans blue extravasation in the ipsilateral hemisphere. Evans blue content in brain tissue was quantified using a standard curve and expressed as Evans blue stain (μg)/weight of brain tissue (g).

### 4.9. Western Blot Analysis of Brain Homogenate after tMCAo

Brain lysates were separated by NuPAGE 4%–12% Bis-Tris gels (Cat# NP0324BOX, Invitrogen) [19], transferred to PVDF membranes, and analyzed by Western blotting using rabbit anti-claudin-5 (Cat# ab131259, Abcam, Cambridge, UK; 1:1000 dilution in Western Antibody Dilution Buffer), goat anti-MPO heavy chain (Cat# AF3667, R&D; 1:400 dilution in Western Antibody Dilution Buffer), and mouse anti-β-actin (Cat# A4700, Sigma-Aldrich; 1:2000 dilution in Western Antibody Dilution Buffer) antibodies. Immunoreactive bands were detected using SuperSignal West Pico PLUS chemiluminescence substrate (Cat# 34080, Thermo), imaged using a luminescent image analyzer LAS-3000 (Fujifilm), and quantified by NIH ImageJ.

### 4.10. Epitope Mapping of LCN2 mAb

Fifty-six 15-mer peptides with an overlap of 12 amino acids were commercially synthesized (Mimotopes, Victoria, Australia) to cover the entire LCN2 protein sequence [63]. These peptides were biotinylated at the *N*-terminus with a 4-amino-acid linker sequence (SGSG) and were included with an amide group at the C terminus. Peptides were prepared to a final stock concentration of 1.4 mM in 40% acetonitrile (Cat# 360457, Sigma-Aldrich). The stock peptides were diluted 1000× in PBS containing 0.1% Tween (PBST) and were analyzed for the reactivity with LCN2 mAb by ELISA according to the supplied protocol (Mimotopes). One hundred μL of each diluted peptide was transferred to the corresponding well position of 96-well plates coated with streptavidin and BSA (Cat# 15125, Pierce), and incubated with agitation for one hour at 20 °C. The plates with immobilized peptides were washed with PBST three times and incubated with LCN2 mAb (1 μg/mL, Cat# MAB18571, R&D) for one hour at 20 °C. After washing, the bound LCN2 mAb was detected by incubation for one hour at 20 °C with HRP-conjugated goat anti-rat IgG2a Secondary Antibody (Cat# PA1-84709, Thermo). The plates were washed with PBST three times and incubated with freshly prepared ABTS (Cat# 37615, Thermo) for 45 min at 20 °C. The bound enzyme was detected by reading the absorbance at 405nm (OD405).

### 4.11. Immunoprecipitation with Competing Epitope Peptides

Dynabeads Protein G (10 µL, Cat# 10007D, Thermo) was incubated with 0.2 μg of LCN2 mAb (Cat# MAB18571, R&D) in 100 µL PBS for 60 min at room temperature [57]. For the competitive interaction between LCN2 mAb and mouse LCN2 protein, LCN2 mAb bound on the Dynabeads was incubated with 0.06 μg of recombinant mouse LCN2 protein (Cat# 1857-LC, R&D) and mouse LCN2 mAb epitope peptide (YNVTSILVRDQDQGCRYWIRT, synthesized by AnaSpec, Fremont, CA, USA) in 10X, 100X, or 1000X molar ratio of LCN2 protein in 100 µL PBST for 16 h at 4 °C. For the competitive interaction between LCN2 mAb and human LCN2 protein, recombinant human LCN2 protein (Cat# 1757-LC, R&D) and human LCN2 mAb epitope peptide (YNVTSVLFRKKKCDYWIRT, synthesized by AnaSpec) were used. The immunoprecipitated mouse or human LCN2 protein was detected by Western blot using goat anti-mouse LCN2 antibody (Cat# AF1857, R&D; 1:1000 dilution in Western Antibody Dilution Buffer) or goat anti-human LCN2 antibody (Cat# AF1757, R&D; 1:1000 dilution in Western Antibody Dilution Buffer), and an EasyBlot anti-goat IgG kit (Cat# GTX228910-01, GeneTex).

### 4.12. Statistical Analysis

Quantitative data were expressed as means ± SEM and analyzed by *t*-tests, one-way ANOVA, and Newman-Keuls post hoc tests, using Prism 5 (GraphPad, La Jolla, CA, USA). *p*-values less than 0.05 were considered to be statistically significant.

## 5. Conclusions

In this study we demonstrated for the first time that treatments with LCN2 mAb reduced ischemic brain injuries after tMCAo, possibly through attenuating the induction of pro-inflammatory cytokines and chemokines, as well as infiltration of neutrophils. These results support the hypothesis that neutralization of LCN2 with specific antibodies may prove useful in treating stroke-reperfusion injury.

## Figures and Tables

**Table 1:** Mice used in the study.

Group	Number of Mice Used for Experiments	Mortality Rate	Number of Mice Used in the Figures
Figure 1 and Figure 2	naive *LCN2^+/+^*: 3	0	3
(staining)	*LCN2^+/+^* tMCAo: 3	0	3
Figure 3B,C	naive *LCN2^+/+^*: 3	0	3
(Immunoprecipitation–Western)	naive *LCN2^−/−^*: 3	0	3
	*LCN2^+/+^* tMCAo: 3	0	3
	*LCN2^−/−^* tMCAo: 3	0	3
Figure 3E,F (ELISA)	*LCN2^+/+^* tMCAo: 10	0	10
	*LCN2^+/+^* tMCAo-Ab: 10	1/10 (10.0%)	9
Figure 3D and Figure 6	naive *LCN2^+/+^*: 6	0	6
(real-time RT-PCR)	naive *LCN2^−/−^*: 6	0	6
	*LCN2^+/+^* tMCAo: 8	2/8 (25.0%)	6
	*LCN2^−/−^* tMCAo: 7	1/7 (14.3%)	6
	*LCN2^+/+^* tMCAo-Ab: 7	1/7 (14.3%)	6
Figure 4A–E (TTC staining)	*LCN2^+/+^* tMCAo: 8	1/8 (12.5%)	7
	*LCN2^+/+^* tMCAo-Ab: 8	1/8 (12.5%)	7
Figure 5A,B (blood–brain barrier (BBB))	*LCN2^+/+^* tMCAo: 6	1/6 (16.6%)	5
	*LCN2^+/+^* tMCAo-Ab: 6	1/6 (16.6%)	5
Figure 5C–F	*LCN2^+/+^* tMCAo: 4	0	4
(BBB–Western)	*LCN2^+/+^* tMCAo-Ab: 4	0	4
Total	108	9/108 (8.3%)	99

**Table 2:** Nucleotide sequences of the primers used in real-time RT-PCR; F, forward primer; R, reverse primer.

Mouse cDNA	Primer Sequences
*LCN2*	F, 5′-ATG TCA CCT CCA TCC TGG TC-3′
	R, 5′-CAC ACT CAC CAC CCA TTC AG-3′
*iNOS*	F, 5′-GCC ACC AAC AAT GGC AAC A-3′
	R, 5′-CGT ACC GGA TGA GCT GTG AAT T-3′
*IL6*	F, 5′-AGT TGC CTT CTT GGG ACT GA-3′
	R, 5′-TCC ACG ATT TCC CAG AGA AC-3′
*CCL2*	F, 5′-TCA GCC AGA TGC AGT TAA CG-3′
	R, 5′-GAT CCT CTT GTA GCT CTC CAG C-3′
*CCL9*	F, 5′-CAA CAG AGA CAA AAG AAG TCC AGA G-3′
	R, 5′-CTT GCT GAT AAA GAT GAT GCC C-3′
*GAPDH*	F, 5′-ACC ACA GTC CAT GCC ATC AC-3′
	R, 5′-CAC CAC CCT GTT GCT GTA GCC-3′

## Supplementary Materials

The following are available online at https://www.mdpi.com/1422-0067/21/17/6253/s1, Figure S1 *C*_t_ values of qPCR results for LCN2 (**A**), iNOS (**C**), IL-6 (**E**), CCL2 (**G**), CCL9 (**I**) and their associated GAPDH’s (**B**,**D**,**F**,**H**,**J**).

## Abbreviations

LCN2	Lipocalin-2
NGAL	neutrophil gelatinase-associated lipocalin
tMCAo	transient middle cerebral artery occlusion
TTC	2,3,5-triphenyltetrazolium chloride
BBB	blood–brain barrier
iNOS	inducible nitric oxide synthase
IL-6	interleukin 6
CCL2	C-C motif chemokine ligand 2
MCP-1	monocyte chemoattractant protein-1
CCL9	C-C motif chemokine ligand 9
MIP-1γ	macrophage inflammatory peptide gamma
tPA	tissue plasminogen activator
ROS	reactive oxygen species
i.p.	intraperitoneal injection
rCBF	regional cerebral blood flow
DAPI	4′, 6-diamidino-2-phenylindole
GAPDH	glyceraldehyde-3-phosphate dehydrogenase
GFAP	glial fibrillary acidic protein
MPO	myeloperoxidase
PDB	Protein Data Bank archive
NMDA	*N*-methyl-d-aspartate
ICAM-1	intercellular adhesion molecule-1
TEER	transendothelial electrical resistance
CCR1	C-C chemokine receptor type 1
