# Supplementary material for: Neutralization of Lipocalin-2 Diminishes Stroke-Reperfusion Injury
Source: Int J Mol Sci. 2020 Aug 29;21(17):6253. doi: 10.3390/ijms21176253 (PMC7503651; doi:10.3390/ijms21176253)
Supplement: Supplementary file 1 [file ijms-21-06253-s001.pdf]

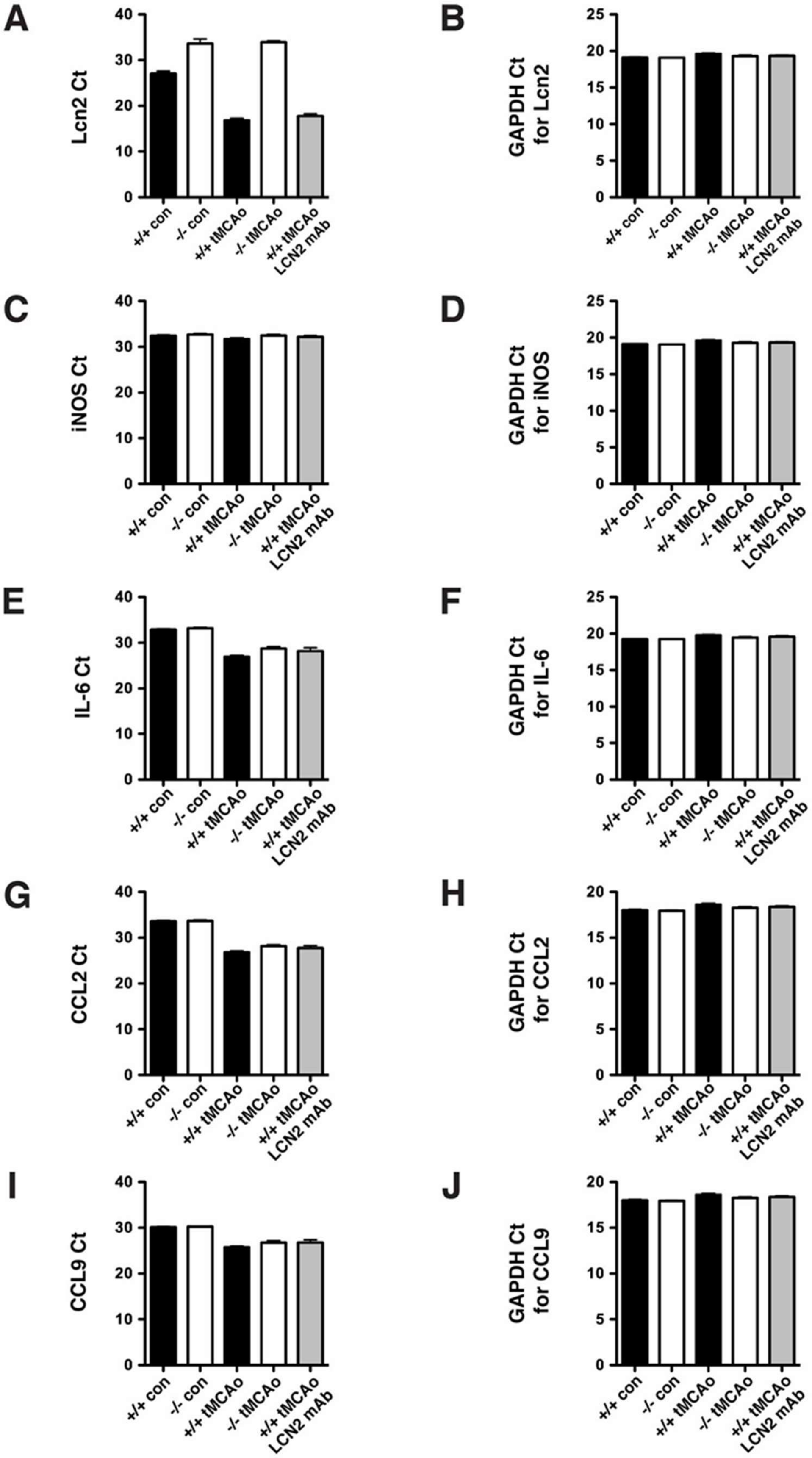

Figure S1. C_t values of qPCR results for LCN2 (**A**), iNOS (**C**), IL-6 (**E**), CCL2 (**G**), CCL9 (**I**) and their associated GAPDH's (**B**,**D**,**F**,**H**,**J**).
